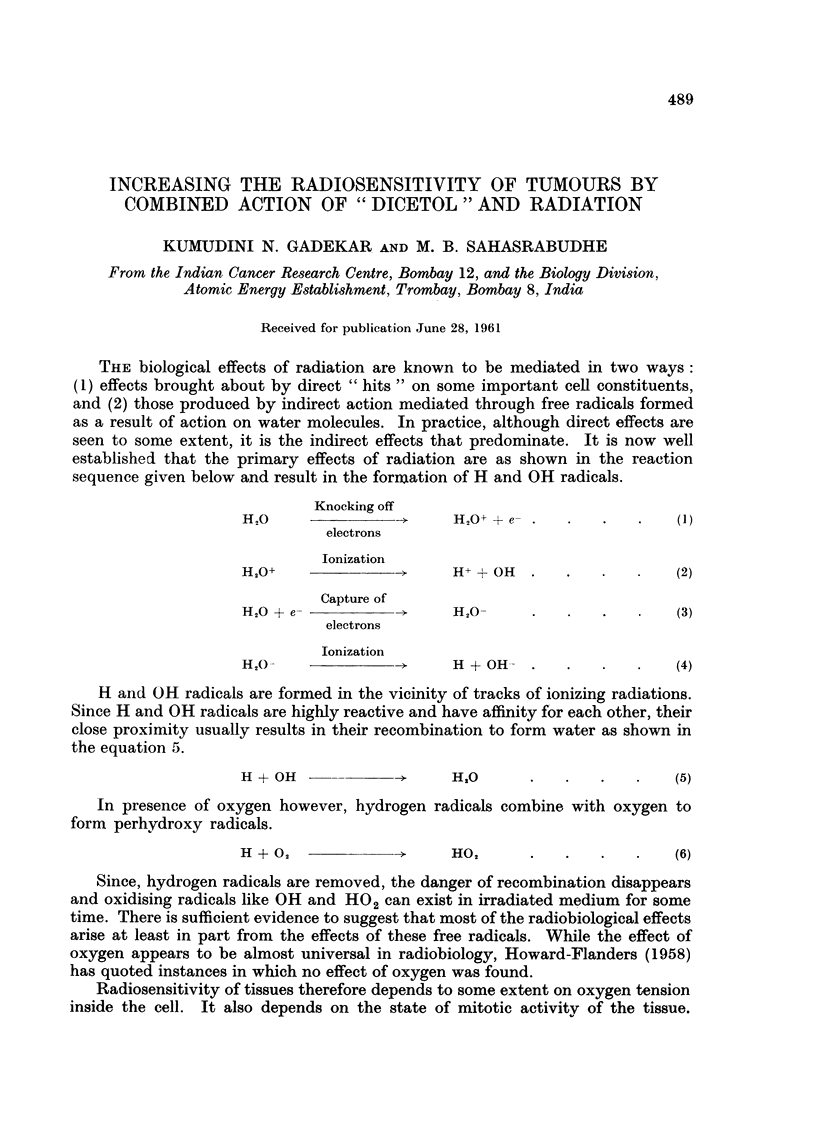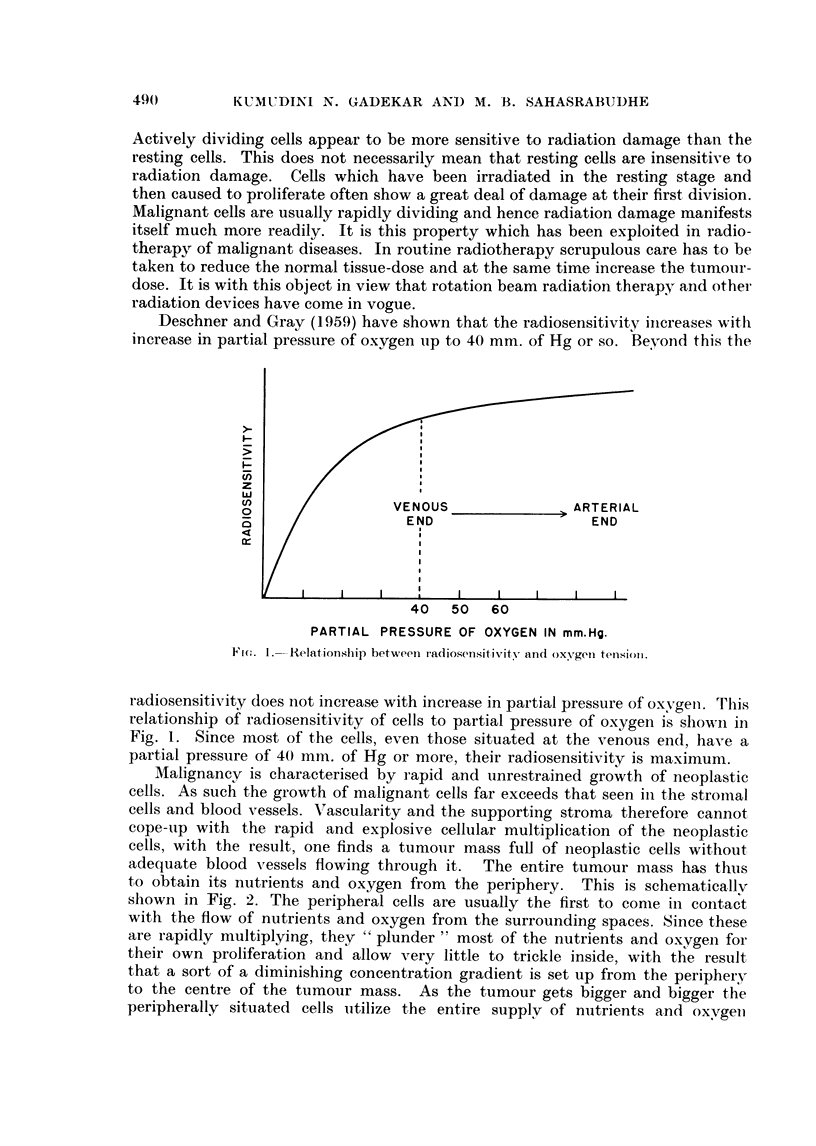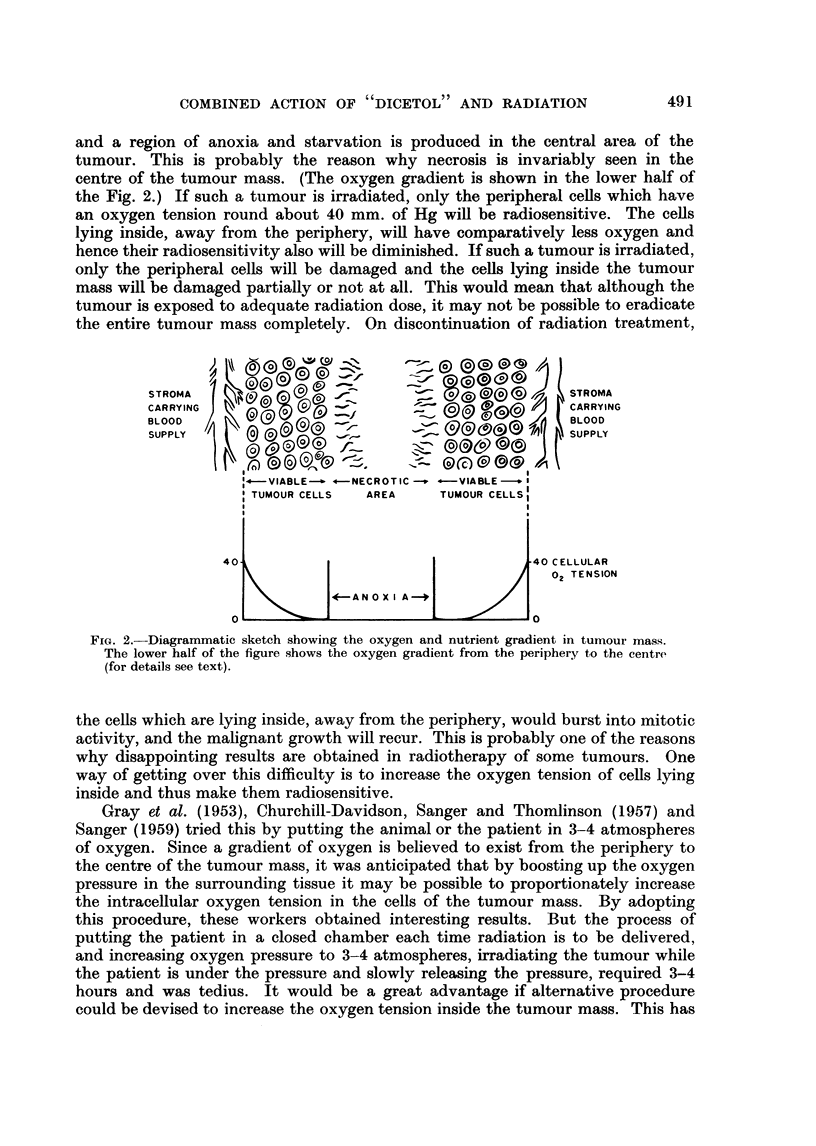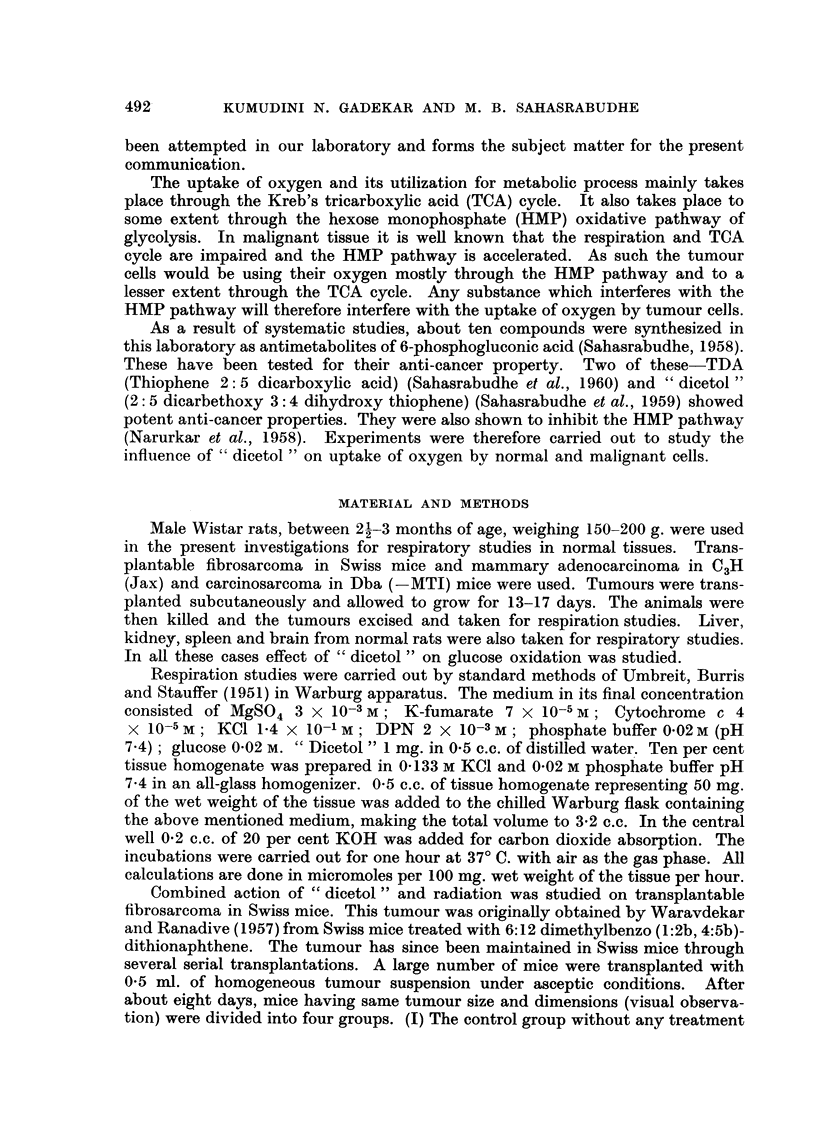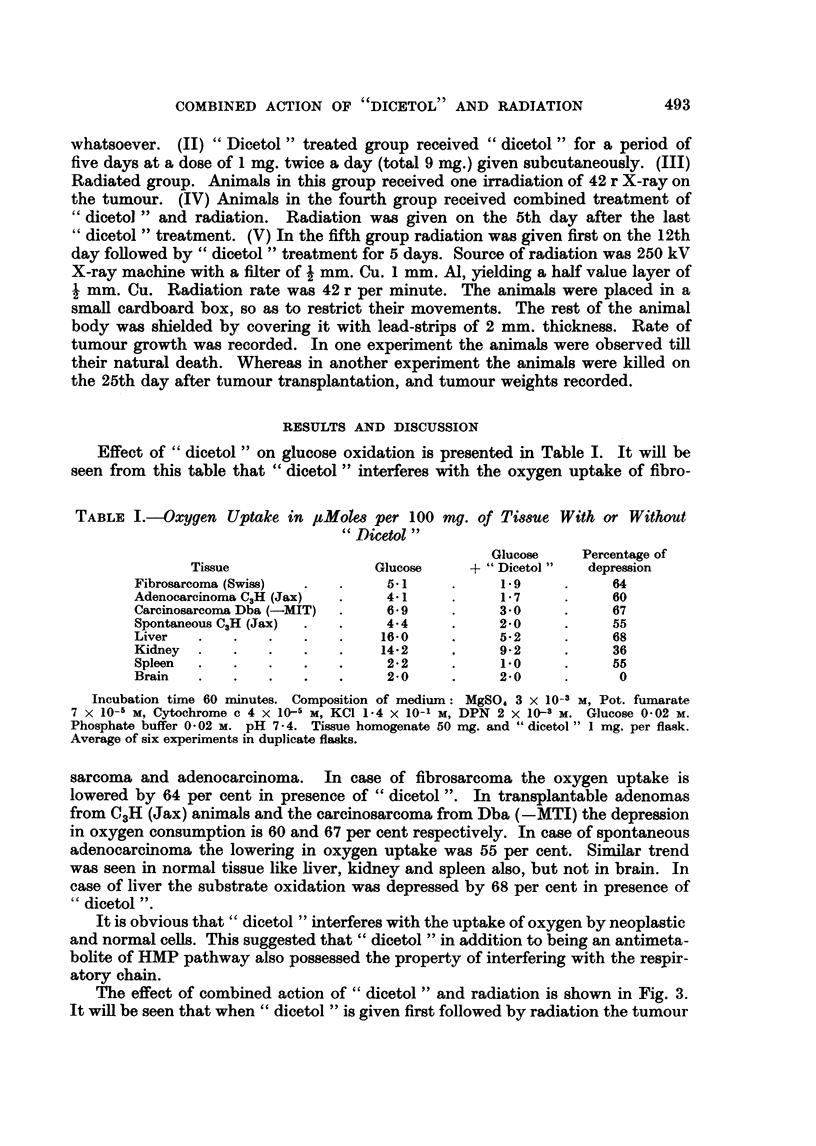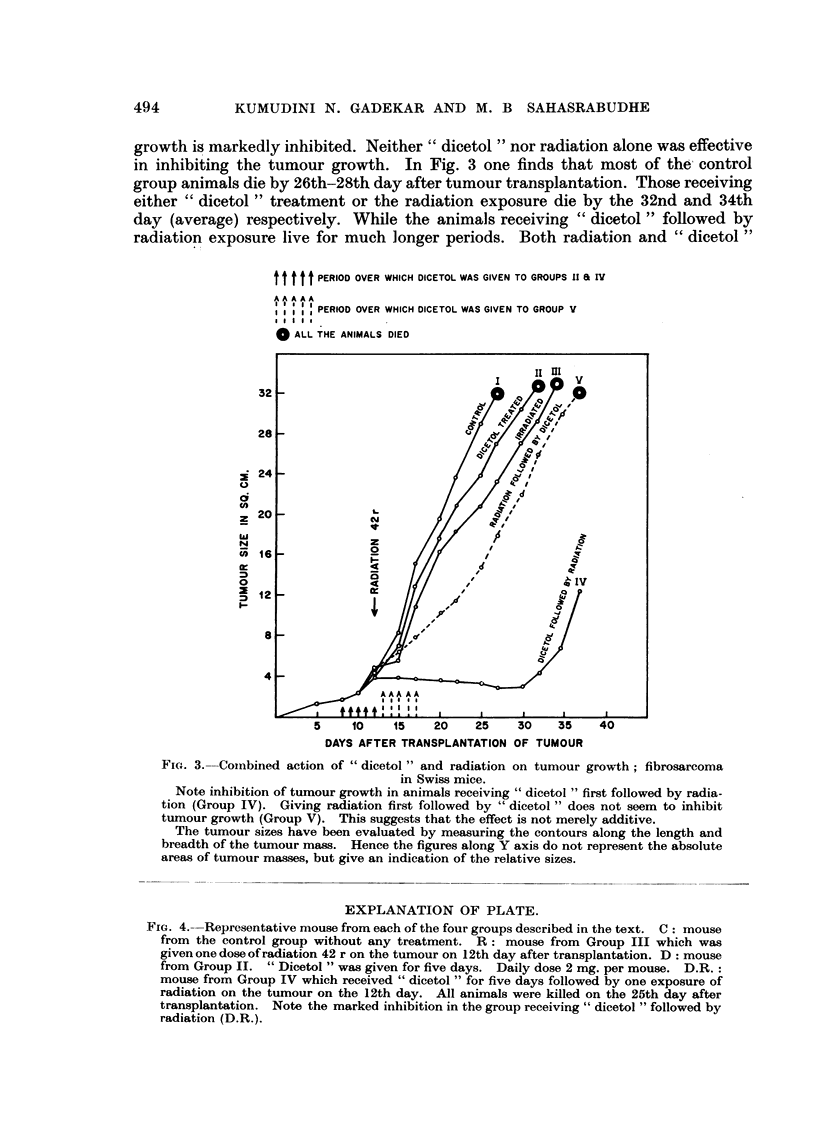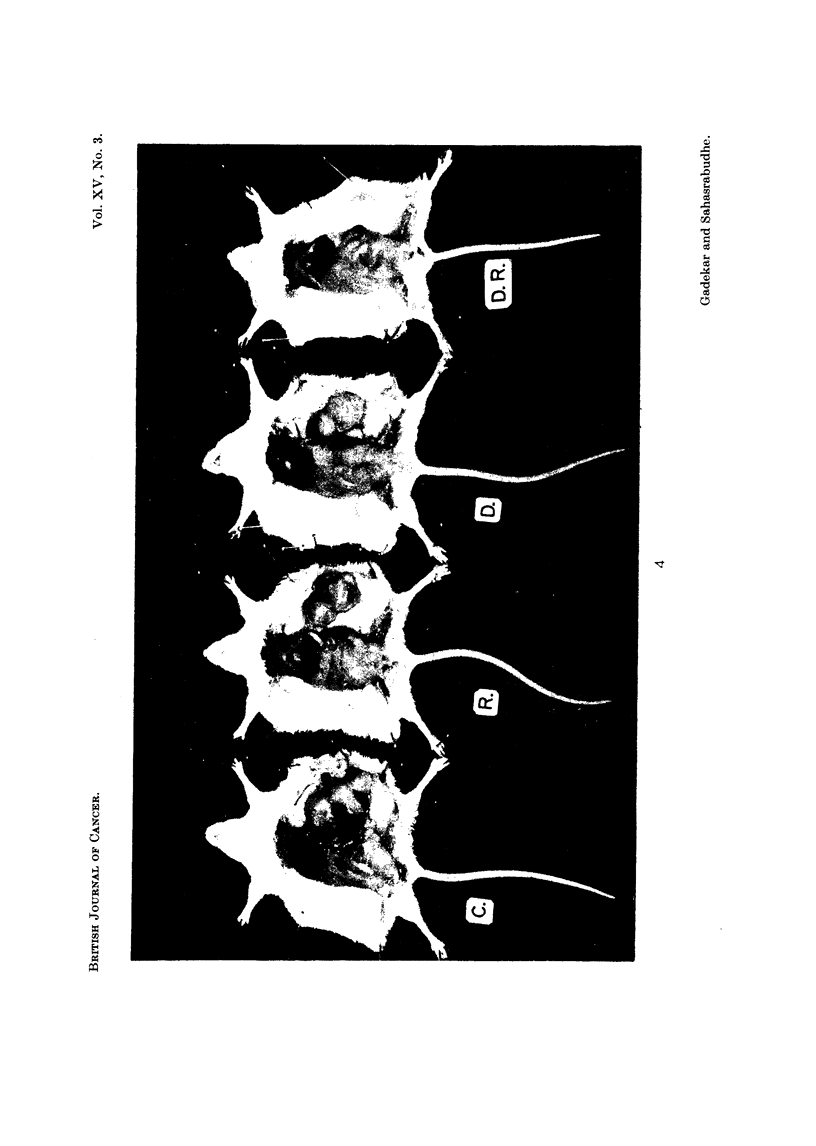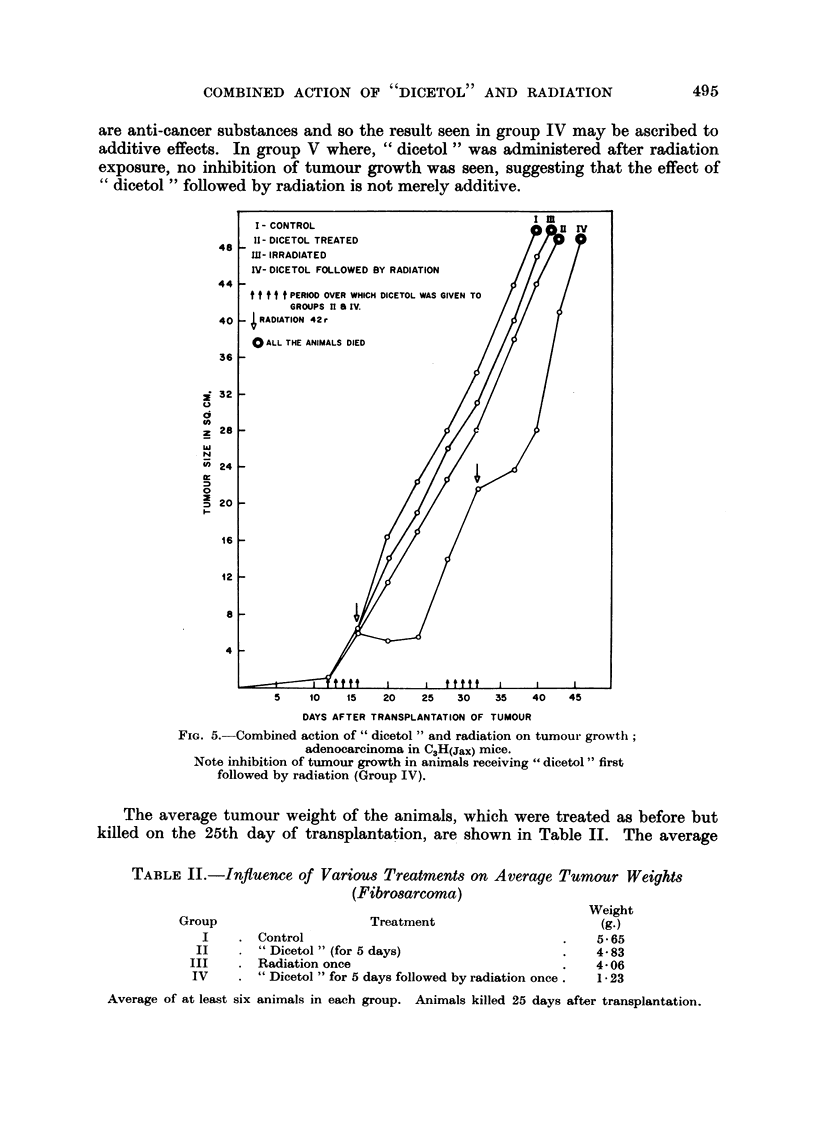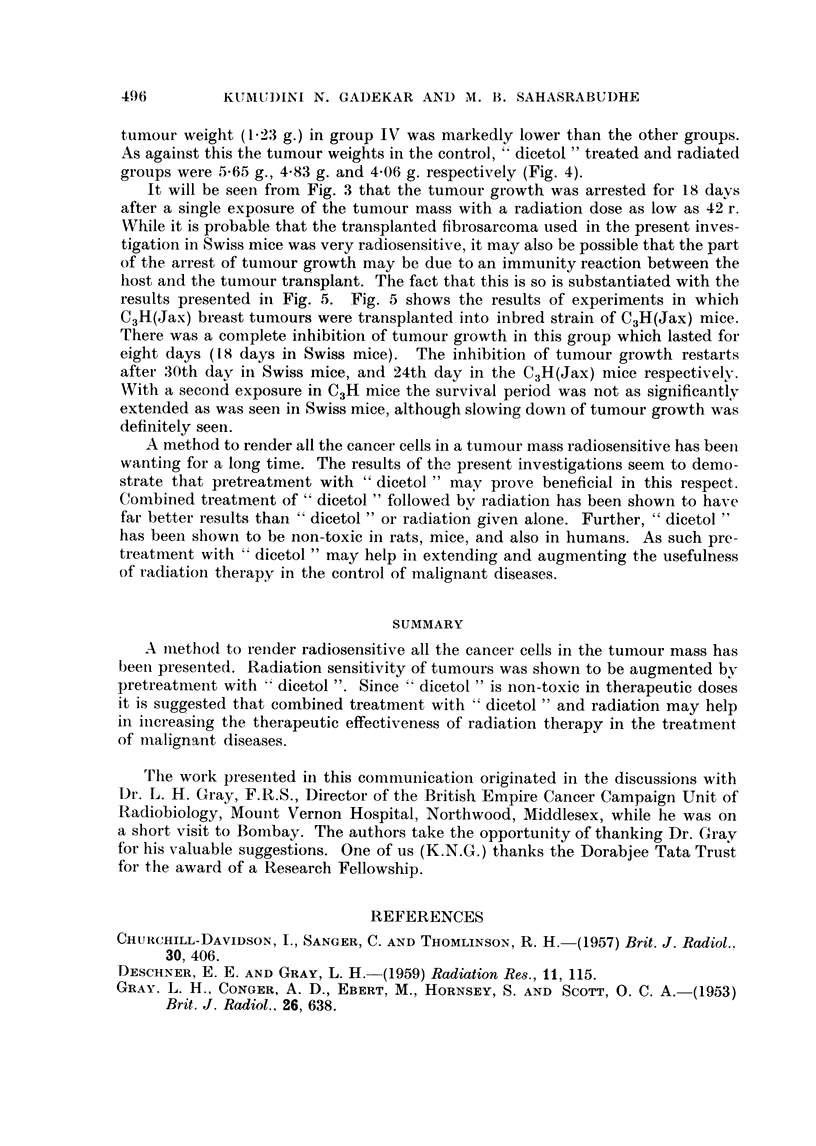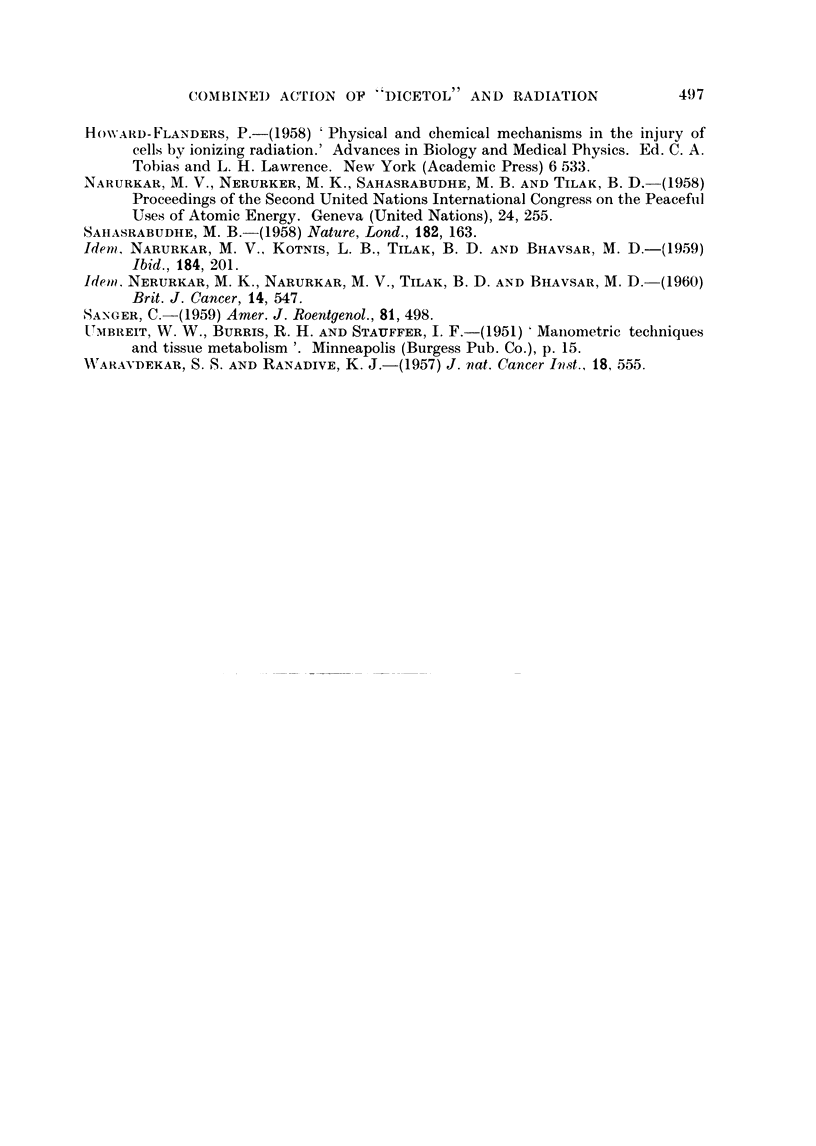# Increasing the Radiosensitivity of Tumours by Combined Action of “Dicetol” and Radiation

**DOI:** 10.1038/bjc.1961.59

**Published:** 1961-09

**Authors:** Kumudini N. Gadekar, M. B. Sahasrabudhe

## Abstract

**Images:**


### INCREASING THE RADIOSENSITIVITY OF TUMOURS BY COMBINED ACTION OF "DICETOL" AND RADIATION

KUMUDINI N. GADEKAR AND M. B. SAHASRABUDHE

*From the Indian Cancer Research Centre, Bombay 12, and the Biology Division, Atomic Energy Establishment, Trombay, Bombay 8, India*

Received for publication June 28, 1961

THE biological effects of radiation are known to be mediated in two ways: (1) effects brought about by direct "hits" on some important cell constituents, and (2) those produced by indirect action mediated through free radicals formed as a result of action on water molecules. In practice, although direct effects are seen to some extent, it is the indirect effects that predominate. It is now well established that the primary effects of radiation are as shown in the reaction sequence given below and result in the formation of H and OH radicals.

$$H_2O \xrightarrow[\text{electrons}]{\text{Knocking off}} H_2O^+ + e^- \quad . \quad . \quad . \quad . \quad (1)$$

$$H_2O^+ \xrightarrow{\text{Ionization}} H^+ + OH \quad . \quad . \quad . \quad . \quad (2)$$

$$H_2O + e^- \xrightarrow[\text{electrons}]{\text{Capture of}} H_2O^- \quad . \quad . \quad . \quad . \quad (3)$$

$$H_2O^- \xrightarrow{\text{Ionization}} H + OH^- \quad . \quad . \quad . \quad . \quad (4)$$

H and OH radicals are formed in the vicinity of tracks of ionizing radiations. Since H and OH radicals are highly reactive and have affinity for each other, their close proximity usually results in their recombination to form water as shown in the equation 5.

$$H + OH \longrightarrow H_2O \quad . \quad . \quad . \quad . \quad (5)$$

In presence of oxygen however, hydrogen radicals combine with oxygen to form perhydroxy radicals.

$$H + O_2 \longrightarrow HO_2 \quad . \quad . \quad . \quad . \quad (6)$$

Since, hydrogen radicals are removed, the danger of recombination disappears and oxidising radicals like OH and $HO_2$ can exist in irradiated medium for some time. There is sufficient evidence to suggest that most of the radiobiological effects arise at least in part from the effects of these free radicals. While the effect of oxygen appears to be almost universal in radiobiology, Howard-Flanders (1958) has quoted instances in which no effect of oxygen was found.

Radiosensitivity of tissues therefore depends to some extent on oxygen tension inside the cell. It also depends on the state of mitotic activity of the tissue.

Actively dividing cells appear to be more sensitive to radiation damage than the resting cells. This does not necessarily mean that resting cells are insensitive to radiation damage. Cells which have been irradiated in the resting stage and then caused to proliferate often show a great deal of damage at their first division. Malignant cells are usually rapidly dividing and hence radiation damage manifests itself much more readily. It is this property which has been exploited in radiotherapy of malignant diseases. In routine radiotherapy scrupulous care has to be taken to reduce the normal tissue-dose and at the same time increase the tumour-dose. It is with this object in view that rotation beam radiation therapy and other radiation devices have come in vogue.

Deschner and Gray (1959) have shown that the radiosensitivity increases with increase in partial pressure of oxygen up to 40 mm. of Hg or so. Beyond this the

FIG. 1.—Relationship between radiosensitivity and oxygen tension.

radiosensitivity does not increase with increase in partial pressure of oxygen. This relationship of radiosensitivity of cells to partial pressure of oxygen is shown in Fig. 1. Since most of the cells, even those situated at the venous end, have a partial pressure of 40 mm. of Hg or more, their radiosensitivity is maximum.

Malignancy is characterised by rapid and unrestrained growth of neoplastic cells. As such the growth of malignant cells far exceeds that seen in the stromal cells and blood vessels. Vascularity and the supporting stroma therefore cannot cope-up with the rapid and explosive cellular multiplication of the neoplastic cells, with the result, one finds a tumour mass full of neoplastic cells without adequate blood vessels flowing through it. The entire tumour mass has thus to obtain its nutrients and oxygen from the periphery. This is schematically shown in Fig. 2. The peripheral cells are usually the first to come in contact with the flow of nutrients and oxygen from the surrounding spaces. Since these are rapidly multiplying, they " plunder " most of the nutrients and oxygen for their own proliferation and allow very little to trickle inside, with the result that a sort of a diminishing concentration gradient is set up from the periphery to the centre of the tumour mass. As the tumour gets bigger and bigger the peripherally situated cells utilize the entire supply of nutrients and oxygen and a region of anoxia and starvation is produced in the central area of the tumour. This is probably the reason why necrosis is invariably seen in the centre of the tumour mass. (The oxygen gradient is shown in the lower half of the Fig. 2.) If such a tumour is irradiated, only the peripheral cells which have an oxygen tension round about 40 mm. of Hg will be radiosensitive. The cells lying inside, away from the periphery, will have comparatively less oxygen and hence their radiosensitivity also will be diminished. If such a tumour is irradiated, only the peripheral cells will be damaged and the cells lying inside the tumour mass will be damaged partially or not at all. This would mean that although the tumour is exposed to adequate radiation dose, it may not be possible to eradicate the entire tumour mass completely. On discontinuation of radiation treatment,

FIG. 2.—Diagrammatic sketch showing the oxygen and nutrient gradient in tumour mass. The lower half of the figure shows the oxygen gradient from the periphery to the centre (for details see text).

the cells which are lying inside, away from the periphery, would burst into mitotic activity, and the malignant growth will recur. This is probably one of the reasons why disappointing results are obtained in radiotherapy of some tumours. One way of getting over this difficulty is to increase the oxygen tension of cells lying inside and thus make them radiosensitive.

Gray *et al.* (1953), Churchill-Davidson, Sanger and Thomlinson (1957) and Sanger (1959) tried this by putting the animal or the patient in 3–4 atmospheres of oxygen. Since a gradient of oxygen is believed to exist from the periphery to the centre of the tumour mass, it was anticipated that by boosting up the oxygen pressure in the surrounding tissue it may be possible to proportionately increase the intracellular oxygen tension in the cells of the tumour mass. By adopting this procedure, these workers obtained interesting results. But the process of putting the patient in a closed chamber each time radiation is to be delivered, and increasing oxygen pressure to 3–4 atmospheres, irradiating the tumour while the patient is under the pressure and slowly releasing the pressure, required 3–4 hours and was tedius. It would be a great advantage if alternative procedure could be devised to increase the oxygen tension inside the tumour mass. This has been attempted in our laboratory and forms the subject matter for the present communication.

The uptake of oxygen and its utilization for metabolic process mainly takes place through the Kreb's tricarboxylic acid (TCA) cycle. It also takes place to some extent through the hexose monophosphate (HMP) oxidative pathway of glycolysis. In malignant tissue it is well known that the respiration and TCA cycle are impaired and the HMP pathway is accelerated. As such the tumour cells would be using their oxygen mostly through the HMP pathway and to a lesser extent through the TCA cycle. Any substance which interferes with the HMP pathway will therefore interfere with the uptake of oxygen by tumour cells.

As a result of systematic studies, about ten compounds were synthesized in this laboratory as antimetabolites of 6-phosphogluconic acid (Sahasrabudhe, 1958). These have been tested for their anti-cancer property. Two of these—TDA (Thiophene 2 : 5 dicarboxylic acid) (Sahasrabudhe *et al.*, 1960) and " dicetol " (2 : 5 dicarbethoxy 3 : 4 dihydroxy thiophene) (Sahasrabudhe *et al.*, 1959) showed potent anti-cancer properties. They were also shown to inhibit the HMP pathway (Narurkar *et al.*, 1958). Experiments were therefore carried out to study the influence of " dicetol " on uptake of oxygen by normal and malignant cells.

### MATERIAL AND METHODS

Male Wistar rats, between $2\frac{1}{2}$–3 months of age, weighing 150–200 g. were used in the present investigations for respiratory studies in normal tissues. Transplantable fibrosarcoma in Swiss mice and mammary adenocarcinoma in $C_3H$ (Jax) and carcinosarcoma in Dba (—MTI) mice were used. Tumours were transplanted subcutaneously and allowed to grow for 13–17 days. The animals were then killed and the tumours excised and taken for respiration studies. Liver, kidney, spleen and brain from normal rats were also taken for respiratory studies. In all these cases effect of " dicetol " on glucose oxidation was studied.

Respiration studies were carried out by standard methods of Umbreit, Burris and Stauffer (1951) in Warburg apparatus. The medium in its final concentration consisted of $MgSO_4$ $3 \times 10^{-3}$ M ; K-fumarate $7 \times 10^{-5}$ M ; Cytochrome *c* $4 \times 10^{-5}$ M ; KCl $1{\cdot}4 \times 10^{-1}$ M ; DPN $2 \times 10^{-3}$ M ; phosphate buffer 0·02 M (pH 7·4) ; glucose 0·02 M. " Dicetol " 1 mg. in 0·5 c.c. of distilled water. Ten per cent tissue homogenate was prepared in 0·133 M KCl and 0·02 M phosphate buffer pH 7·4 in an all-glass homogenizer. 0·5 c.c. of tissue homogenate representing 50 mg. of the wet weight of the tissue was added to the chilled Warburg flask containing the above mentioned medium, making the total volume to 3·2 c.c. In the central well 0·2 c.c. of 20 per cent KOH was added for carbon dioxide absorption. The incubations were carried out for one hour at 37° C. with air as the gas phase. All calculations are done in micromoles per 100 mg. wet weight of the tissue per hour.

Combined action of " dicetol " and radiation was studied on transplantable fibrosarcoma in Swiss mice. This tumour was originally obtained by Waravdekar and Ranadive (1957) from Swiss mice treated with 6:12 dimethylbenzo (1:2b, 4:5b)-dithionaphthene. The tumour has since been maintained in Swiss mice through several serial transplantations. A large number of mice were transplanted with 0·5 ml. of homogeneous tumour suspension under asceptic conditions. After about eight days, mice having same tumour size and dimensions (visual observation) were divided into four groups. (I) The control group without any treatment whatsoever. (II) " Dicetol " treated group received " dicetol " for a period of five days at a dose of 1 mg. twice a day (total 9 mg.) given subcutaneously. (III) Radiated group. Animals in this group received one irradiation of 42 r X-ray on the tumour. (IV) Animals in the fourth group received combined treatment of " dicetol " and radiation. Radiation was given on the 5th day after the last " dicetol " treatment. (V) In the fifth group radiation was given first on the 12th day followed by " dicetol " treatment for 5 days. Source of radiation was 250 kV X-ray machine with a filter of ½ mm. Cu. 1 mm. Al, yielding a half value layer of ½ mm. Cu. Radiation rate was 42 r per minute. The animals were placed in a small cardboard box, so as to restrict their movements. The rest of the animal body was shielded by covering it with lead-strips of 2 mm. thickness. Rate of tumour growth was recorded. In one experiment the animals were observed till their natural death. Whereas in another experiment the animals were killed on the 25th day after tumour transplantation, and tumour weights recorded.

### RESULTS AND DISCUSSION

Effect of " dicetol " on glucose oxidation is presented in Table I. It will be seen from this table that " dicetol " interferes with the oxygen uptake of fibro-

TABLE I.—*Oxygen Uptake in µMoles per* 100 *mg. of Tissue With or Without " Dicetol "*

| Tissue | Glucose | Glucose + " Dicetol " | Percentage of depression |
|---|---|---|---|
| Fibrosarcoma (Swiss) | 5·1 | 1·9 | 64 |
| Adenocarcinoma $C_3H$ (Jax) | 4·1 | 1·7 | 60 |
| Carcinosarcoma Dba (—MIT) | 6·9 | 3·0 | 67 |
| Spontaneous $C_3H$ (Jax) | 4·4 | 2·0 | 55 |
| Liver | 16·0 | 5·2 | 68 |
| Kidney | 14·2 | 9·2 | 36 |
| Spleen | 2·2 | 1·0 | 55 |
| Brain | 2·0 | 2·0 | 0 |

Incubation time 60 minutes. Composition of medium: $MgSO_4$ $3 \times 10^{-3}$ M, Pot. fumarate $7 \times 10^{-5}$ M, Cytochrome c $4 \times 10^{-5}$ M, KCl $1·4 \times 10^{-1}$ M, DPN $2 \times 10^{-3}$ M. Glucose 0·02 M. Phosphate buffer 0·02 M. pH 7·4. Tissue homogenate 50 mg. and " dicetol " 1 mg. per flask. Average of six experiments in duplicate flasks.

sarcoma and adenocarcinoma. In case of fibrosarcoma the oxygen uptake is lowered by 64 per cent in presence of " dicetol ". In transplantable adenomas from $C_3H$ (Jax) animals and the carcinosarcoma from Dba (—MTI) the depression in oxygen consumption is 60 and 67 per cent respectively. In case of spontaneous adenocarcinoma the lowering in oxygen uptake was 55 per cent. Similar trend was seen in normal tissue like liver, kidney and spleen also, but not in brain. In case of liver the substrate oxidation was depressed by 68 per cent in presence of " dicetol ".

It is obvious that " dicetol " interferes with the uptake of oxygen by neoplastic and normal cells. This suggested that " dicetol " in addition to being an antimetabolite of HMP pathway also possessed the property of interfering with the respiratory chain.

The effect of combined action of " dicetol " and radiation is shown in Fig. 3. It will be seen that when " dicetol " is given first followed by radiation the tumour growth is markedly inhibited. Neither " dicetol " nor radiation alone was effective in inhibiting the tumour growth. In Fig. 3 one finds that most of the control group animals die by 26th–28th day after tumour transplantation. Those receiving either " dicetol " treatment or the radiation exposure die by the 32nd and 34th day (average) respectively. While the animals receiving " dicetol " followed by radiation exposure live for much longer periods. Both radiation and " dicetol "

Fig. 3.—Combined action of " dicetol " and radiation on tumour growth ; fibrosarcoma in Swiss mice.

Note inhibition of tumour growth in animals receiving " dicetol " first followed by radiation (Group IV). Giving radiation first followed by " dicetol " does not seem to inhibit tumour growth (Group V). This suggests that the effect is not merely additive.

The tumour sizes have been evaluated by measuring the contours along the length and breadth of the tumour mass. Hence the figures along Y axis do not represent the absolute areas of tumour masses, but give an indication of the relative sizes.

---

### EXPLANATION OF PLATE.

Fig. 4.—Representative mouse from each of the four groups described in the text. C : mouse from the control group without any treatment. R : mouse from Group III which was given one dose of radiation 42 r on the tumour on 12th day after transplantation. D : mouse from Group II. " Dicetol " was given for five days. Daily dose 2 mg. per mouse. D.R. : mouse from Group IV which received " dicetol " for five days followed by one exposure of radiation on the tumour on the 12th day. All animals were killed on the 25th day after transplantation. Note the marked inhibition in the group receiving " dicetol " followed by radiation (D.R.).

4

Gadekar and Sahasrabudhe.

are anti-cancer substances and so the result seen in group IV may be ascribed to additive effects. In group V where, " dicetol " was administered after radiation exposure, no inhibition of tumour growth was seen, suggesting that the effect of " dicetol " followed by radiation is not merely additive.

FIG. 5.—Combined action of " dicetol " and radiation on tumour growth ; adenocarcinoma in $C_3H$(Jax) mice.
Note inhibition of tumour growth in animals receiving " dicetol " first followed by radiation (Group IV).

The average tumour weight of the animals, which were treated as before but killed on the 25th day of transplantation, are shown in Table II. The average

TABLE II.—*Influence of Various Treatments on Average Tumour Weights (Fibrosarcoma)*

| Group | Treatment | Weight (g.) |
|---|---|---|
| I | Control | 5·65 |
| II | " Dicetol " (for 5 days) | 4·83 |
| III | Radiation once | 4·06 |
| IV | " Dicetol " for 5 days followed by radiation once | 1·23 |

Average of at least six animals in each group. Animals killed 25 days after transplantation.

tumour weight (1·23 g.) in group IV was markedly lower than the other groups. As against this the tumour weights in the control, " dicetol " treated and radiated groups were 5·65 g., 4·83 g. and 4·06 g. respectively (Fig. 4).

It will be seen from Fig. 3 that the tumour growth was arrested for 18 days after a single exposure of the tumour mass with a radiation dose as low as 42 r. While it is probable that the transplanted fibrosarcoma used in the present investigation in Swiss mice was very radiosensitive, it may also be possible that the part of the arrest of tumour growth may be due to an immunity reaction between the host and the tumour transplant. The fact that this is so is substantiated with the results presented in Fig. 5. Fig. 5 shows the results of experiments in which $C_3H$(Jax) breast tumours were transplanted into inbred strain of $C_3H$(Jax) mice. There was a complete inhibition of tumour growth in this group which lasted for eight days (18 days in Swiss mice). The inhibition of tumour growth restarts after 30th day in Swiss mice, and 24th day in the $C_3H$(Jax) mice respectively. With a second exposure in $C_3H$ mice the survival period was not as significantly extended as was seen in Swiss mice, although slowing down of tumour growth was definitely seen.

A method to render all the cancer cells in a tumour mass radiosensitive has been wanting for a long time. The results of the present investigations seem to demostrate that pretreatment with " dicetol " may prove beneficial in this respect. Combined treatment of " dicetol " followed by radiation has been shown to have far better results than " dicetol " or radiation given alone. Further, " dicetol " has been shown to be non-toxic in rats, mice, and also in humans. As such pretreatment with " dicetol " may help in extending and augmenting the usefulness of radiation therapy in the control of malignant diseases.

### SUMMARY

A method to render radiosensitive all the cancer cells in the tumour mass has been presented. Radiation sensitivity of tumours was shown to be augmented by pretreatment with " dicetol ". Since " dicetol " is non-toxic in therapeutic doses it is suggested that combined treatment with " dicetol " and radiation may help in increasing the therapeutic effectiveness of radiation therapy in the treatment of malignant diseases.

The work presented in this communication originated in the discussions with Dr. L. H. Gray, F.R.S., Director of the British Empire Cancer Campaign Unit of Radiobiology, Mount Vernon Hospital, Northwood, Middlesex, while he was on a short visit to Bombay. The authors take the opportunity of thanking Dr. Gray for his valuable suggestions. One of us (K.N.G.) thanks the Dorabjee Tata Trust for the award of a Research Fellowship.

### REFERENCES

Churchill-Davidson, I., Sanger, C. and Thomlinson, R. H.—(1957) *Brit. J. Radiol.*, **30**, 406.

Deschner, E. E. and Gray, L. H.—(1959) *Radiation Res.*, **11**, 115.

Gray, L. H., Conger, A. D., Ebert, M., Hornsey, S. and Scott, O. C. A.—(1953) *Brit. J. Radiol.*, **26**, 638.

HOWARD-FLANDERS, P.—(1958) ' Physical and chemical mechanisms in the injury of cells by ionizing radiation.' Advances in Biology and Medical Physics. Ed. C. A. Tobias and L. H. Lawrence. New York (Academic Press) 6 533.

NARURKAR, M. V., NERURKER, M. K., SAHASRABUDHE, M. B. AND TILAK, B. D.—(1958) Proceedings of the Second United Nations International Congress on the Peaceful Uses of Atomic Energy. Geneva (United Nations), 24, 255.

SAHASRABUDHE, M. B.—(1958) *Nature, Lond.*, **182**, 163.

*Idem*, NARURKAR, M. V., KOTNIS, L. B., TILAK, B. D. AND BHAVSAR, M. D.—(1959) *Ibid.*, **184**, 201.

*Idem*, NERURKAR, M. K., NARURKAR, M. V., TILAK, B. D. AND BHAVSAR, M. D.—(1960) *Brit. J. Cancer*, **14**, 547.

SANGER, C.—(1959) *Amer. J. Roentgenol.*, **81**, 498.

UMBREIT, W. W., BURRIS, R. H. AND STAUFFER, I. F.—(1951) ' Manometric techniques and tissue metabolism '. Minneapolis (Burgess Pub. Co.), p. 15.

WARAVDEKAR, S. S. AND RANADIVE, K. J.—(1957) *J. nat. Cancer Inst.*, **18**, 555.